# First isolation and full-length genome analysis of a new RNA virus, Wenzhou sobemo-like virus 4 from *Culex tritaeniorhynchus*

**DOI:** 10.1080/21505594.2025.2539210

**Published:** 2025-08-04

**Authors:** Yuli Zhang, Yu Bi, Xiaohui Zou, Zhen Wu, Hengyi Sun, Jian Song, Guoyu Niu

**Affiliations:** a Shandong Second Medical University, Weifang, China; b National Institute for Viral Disease Control and Prevention, Chinese Center for Disease Control and Prevention, Beijing, China

**Keywords:** Wenzhou sobemo-like virus 4, mosquitoes, growth characteristics, genome, Shandong Province

## Abstract

Arboviruses are a major public health concern. Wenzhou sobemo-like virus 4 (WZSLV4) is a recently identified single-stranded RNA mosquito-borne virus. In this study, 3608 mosquitoes were collected from Shandong Province, China, and divided into 58 pools according to species and sex. qRT-PCR and nested PCR were used to confirm the presence of WZSLV4. Cell culture was used to isolate the virus, and the growth characteristics of WZSLV4 were observed. WZSLV4 was detected in *Aedes albopictus* (12.07 %, 7/58) and *Culex tritaeniorhynchus* (18.97 %,11/58). WZSLV4 replicated effectively in C6/36 cells but grew slowly in Vero and BHK cells without causing cytopathic effect (CPE). Phylogenetic analysis demonstrated that WZSLV4 was closely related to the Guangzhou sobemo-like virus and Sichuan mosquito sobemo-like virus, which belongs to the family *Solemoviridae*. Pairwise distance analysis indicated that 11 partially amplified sequences shared a high nucleotide identity. The objective of this study was to isolate and characterize WZSLV4 from mosquito populations in Shandong Province, China, and to evaluate its growth characteristics in different cell lines to better understand its biological properties and potential pathogenicity. To our knowledge, this is the first report on the isolation of WZSLV4 from mosquitoes, along with a description of the biogenic characterization of WZSLV4 in different cell lines. Nevertheless, further studies are needed to assess the potential pathogenic risks in mammals and humans.

## GRAPHICAL ABSTRACT

**Figure:**
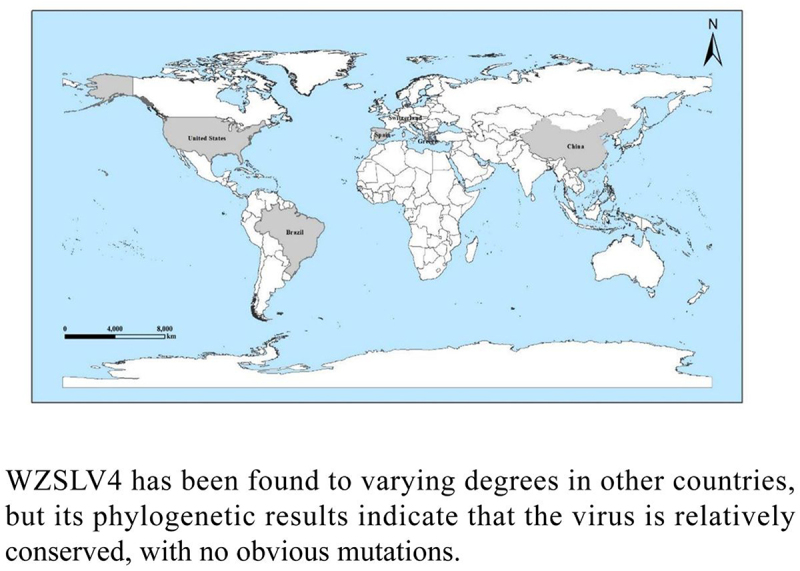

## Introduction

Mosquitoes are pervasive insects that are classified into approximately 3,500 species and further grouped into 41 genera [1]. The commonly studied mosquito species are *Culex tritaeniorhynchus*, *Anopheles sinensis*, *Cx quinquefasciatus*, *Aedes albopictus*, and *Armigeres subalbatus*. These mosquitoes carry numerous causative agents, such as Plasmodium and various viruses. Infections caused by these mosquito-borne pathogens result in over half a million deaths annually [2]. The control of arthropod borne viruses is a primary concern for global public health. However, this challenge is compounded by widespread insecticide resistance in insect vectors and the continued growth of resistance in urban environments [3].

Recently, due to the increasing global prevalence of arboviruses, people have gained a new perspective on arboviral diseases. Moreover, there is a risk of spreading arboviruses, particularly among highly exposed populations [4,5] and in the densely populated African region [6]. Generally, these viruses are divided into two types: mosquito-specific and mosquito-borne viruses. Mosquito-specific viruses, also known as insect-specific viruses (ISVs), not only infect mosquitoes naturally but also replicate in mosquito cells in vitro. However, these viruses do not replicate in vertebrate cells or infect humans or other vertebrates [7]. In contrast, mosquito-borne viruses can replicate in mosquitoes and be transmitted biologically to vertebrate animals, infecting vertebrate cells and altering cell morphology [8]. The majority of mosquito-borne viruses are pathogenic viruses that cause outbreaks of disease in human and animal populations, such as dengue virus (DENV) [9], chikungunya virus (CHIKV) [10], yellow fever virus (YFV) [11], Zika virus (ZIKV) [12], and Akabane virus (AKV) [13]. Arbovirus are highly adaptable to new vectors, which may increase their virulence and transmission capacity [14]. Some simple genetic changes can affect the interaction between the virus and host, leading to indeterminate but potentially significant consequences [15].

Wenzhou sobemo-like virus 4 (WZSLV4) is a positive-sense, single-stranded RNA virus found in *Culicidae* collected from China in 2013 and described in 2016 [16]. Subsequently, WZSLV4 was detected in other locations in *Aedes albopictus*, including Spain in 2015 [17], the United States in 2017 [18], Greece [19], Switzerland [20] and Brazil [21] in 2019, Guangzhou, China in 2023 [22], India in 2024 [23]. To date, the isolation of WZSLV4 from mosquitoes has not been reported in China or abroad and its pathogenicity in mammals and humans has not been demonstrated. In this study, we aimed to isolate WZSLV4 from mosquitoes in Shandong Province, China, characterize its replication dynamics in mosquito (C6/36) and mammalian (Vero, BHK-21) cells, and analyse its complete genome to define its phylogenetic position within the Solemoviridae family. Through comprehensive field collection, 3,608 mosquitoes were collected from Shandong Province, and WZSLV4 was detected in both Culex tritaeniorhynchus and Aedes albopictus, revealing that the virus was widely distributed in eastern China. Additionally, one live viral strain, 18-LZ-22, was successfully isolated from Culex tritaeniorhynchus, which laid a significant foundation for the study of the biological characteristics of WZSLV4. Phylogenetic analysis suggested that this virus was evolutionarily conserved and clustered with the Guangzhou sobemo-like virus and Sichuan mosquito sobemo-like virus. Furthermore, the growth characteristics of WZSLV4 in different cell types were observed to determine its pathogenicity in mammals. Our study enriches the relevant knowledge on WZSLV4 and confirmed its widespread existence in eastern China for the first time.

## Methods

### Mosquito collecting and nucleic acid extraction

In June and July 2018, mosquitoes were collected using light traps baited with CO_2_ from the pens of sheep in Yantai and Weifang, Shandong Province, China. After morphological identification using a stereomicroscope, the collected samples were classified based on external features, species, and sex. Subsequently, mosquitoes were divided into pools of approximately 55–60 individuals and placed in microcentrifuge tubes. The mosquitoes were homogenized in 1000 μL of DMEM using a tissue homogenizer (Qiagen, Germany). After mixing and centrifugation at 4°C and 10,000 ×g for 5 min, RNA was extracted from the clarified supernatant of the mosquito homogenates using the TIANamp RNA extraction kit (Tiangen, China) following the manufacturer’s instructions.

### PCR for detection of WZSLV4

The QIAGEN one-step RT-PCR kit was used for qRT-PCR. A template of 5 μL mosquito nucleic acid, WZSLV4-F (GAC ACT TTG ATA GAC CGC ATT CTG T), WZSLV4-R (CAA ACA CGG CGT TTC ACC TAC T), WZSLV4-P (FAM-CCT GGA TAG CCC GAG CAC AGT TGA GTA C-BHQ1), and double-distilled water were mixed into the system. The reaction conditions were as follows: 50°C for 30 min, 95°C for 15 min, 94°C for 30 s, 55°C for 23 s, and 72°C for 1 min. Forty cycles of amplification were performed, followed by a final step at 72°C for 10 min and storage at 4°C. All positive specimens from the primary screening were further confirmed by nested PCR using the primer sets Out-F (ATG ACT CGG GAA ACT TGC TGA G), Out-R (ACC CGC TCT TCA TCA CTC CTT), In-F (GTC CGA CGG GAG TTT GTA CG), and In-R (GAA CCG AAA CAC TGC GTC CT). The amplified products were separated by electrophoresis in a 1% agarose gel, and the electrophoresis products were then visualized by SYBR Safe (Thermo Fisher Scientific) and subjected to direct Sanger sequencing (Shanghai Sangon Biotechnology Co., Shanghai, China). The complete genome of WZSLV4 was sequenced, and the sequence was deposited in GenBank under accession number ON918610. The partial sequences of WZSLV4 obtained from nested PCR were deposited in the GenBank database and assigned accession numbers (ON952537-ON952547).

### Cell culture

The mosquito (*Aedes albopictus*) cell line, C6/36, was used to isolate the virus. Two mammalian cell lines, Vero and BHK-21, were used to analyse the growth characteristics of the virus. C6/36 cells were cultivated in SIM SF containing 10% foetal bovine serum (FBS) and 1% penicillin and streptomycin and maintained at 30°C in 5% CO_2_. The BHK-21 and Vero cells were cultured in DMEM containing 10% FBS and 1% penicillin and streptomycin, which were maintained at 37°C in 5% CO_2_. All three cell lines were gifted by the Hong Tao Laboratory of National Institute for Viral Disease Control and Prevention, Chinese Center for Disease Control and Prevention, and were preserved by our team.

### Viral isolation and growth curves

The homogenate, which was positive for WZSLV4, was centrifuged, and the 50 μL supernatant was passed through a sterile 0.22-μm filter (Millipore Sigma, Burlington, MA, USA). The filtrate was then inoculated onto monolayers of approximately 70% confluent C6/36 cells in 6-well culture plates. The plates were incubated for 1 h at 30°C in an atmosphere of 5% CO_2_ to allow virus invasion. After adding 1000 μL of fresh medium, cells were incubated under the same conditions for approximately 10–12 days. After blind-passaging three times, the supernatant was harvested and stored at − 80°C until analysis.

Subsequently, the virus was inoculated into T25 of cultured C6/36, BHK-21 and Vero cells of appropriate density, and incubated for 6 h. The inoculation was then discarded and the cells were washed twice with PBS, followed by replenishment of 5 mL of cell culture medium. And then cell supernatant was collected every other day for testing, with 160 µL of supernatant taken at 6 hours, 2, 4, 6, 8, 10, and 12 days after virus inoculation. The supernatants were centrifuged and filtered, and the resulting material set aside at −80°C. Nucleic acid extraction and qRT-PCR were performed after 12 days [24]. The titre of the WZSLV 4 strain 18-LZ-22 culture supernatants infected C6/36 cells was determined by plaque forming unit (PFU) quantification. The virus preparations were diluted in a 10-fold dilution series from 10^9^ to 10^1^ PFUs/mL. Genomic RNAs were extracted from 140 µL of these diluted viral preparations for qRT-PCR detection, with the aim of generating calibration curves.

### Virus purification and electron microscopy

The virus was purified prior to electron microscopy. The virus culture supernatant, which was inoculated onto monolayers of C6/36 cells, was collected, clarified, and centrifuged at 5000 ×g at 4°C for 30 min to remove cell debris. Ultracentrifugation was performed by adding 2 mL of harvested virus supernatant to the bottom of the ultracentrifugation tube and then carefully adding 4 mL of 20% (w/v) sucrose and phosphate-buffered saline (PBS) to the tube to remove impurities. At 4°C, 3.5 h of ultracentrifugation (Beckman Coulter diagnostics) was performed in a Type 70 Ti rotor at 40,000 ×g. After the completion of the reaction, the supernatant was discarded. Then, 100 µL of PBS was added to the sediment at the bottom of the tube, and the sediment was resuspended. Pure virus particles were added to a Formvar carbon-coated copper grid for 10 min and negatively stained for 2–3 min with 2% phosphotungstic acid (PTA). The samples were examined using an electron microscope (FEI TECNAI 12).

### Genome sequencing

The AllPrep DNA/RNA mini Kit (Qiagen) was used to extract total DNA and RNA from the sample according to the manufacturer’s instructions. Total RNA was subjected to library construction following a standard protocol provided by Illumina Inc. (San Diego, CA, USA). In brief, after extracting total RNA, ribosomal RNA is removed from the sample, and the remaining RNA is fragmented, reverse transcribed, ligated with adapters, and purified. Subsequently, quality checks and trimming are performed, ORF prediction processing is carried out, and the sequences are assembled de novo into contigs ( > 200 bp) using MegaHit. The resulting contigs are translated and compared with the NCBI/NR reference database. At the same time, Sanger sequencing primers are designed based on the results of deep sequencing. These procedures align with our prior methodology for viral genome sequencing [25]. Contigs were specifically compared against viral protein sequences in the NCBI/NR database with an E-value threshold of 1e-5 and minimum identity of 30%.

### Data analyses

The pooled infection rate was calculated as follows: pooled infection rate = positive specimen pool number/total number of processed specimen pools. Statistical analyses were performed using Prism software version 5.00. Fisher’s exact test was performed to evaluate statistical differences in the positive rate. Statistical significance was set at *p* < 0.05. Growth curves were generated using a two-way ANOVA with Bonferroni post-hoc tests in GraphPad Prism 9. All viral sequence alignments and pairwise divergence analyses were conducted using MegAlign, implemented in the DNASTAR software package (Lasergene, USA). Phylogenetic analyses of WZSLV4-related sequences were conducted using nucleotide sequences and partial amino acid sequences obtained from the GenBank database. Phylogenetic trees were generated by MEGA 7 using the neighbour-joining method with 1,000 replicates, with bootstrap values > 70% considered statistically significant.

### Institutional review board statement

Sample collection and experimental procedures were approved by the Ethics Committee of Shandong Second Medical University (2018WZV169).

## Results

### Mosquito specimens

**Figure 1.**
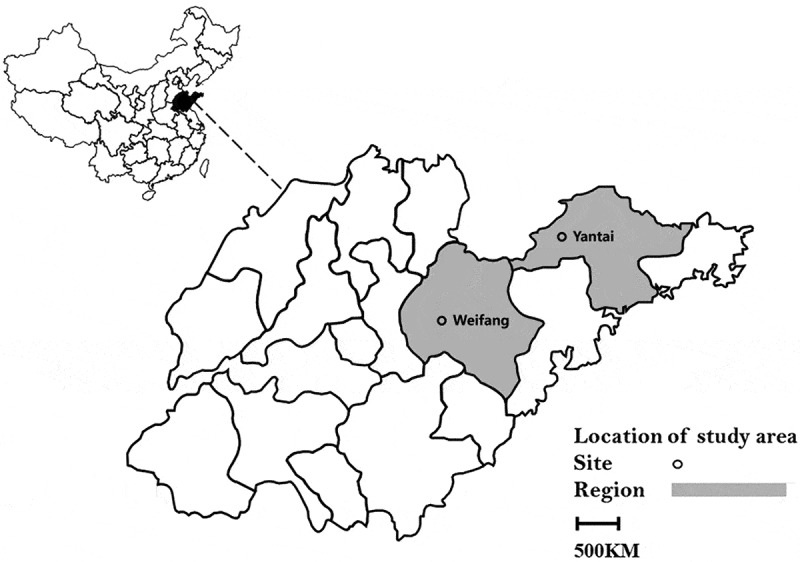
Geographic location of mosquito collection sites in Shandong Province, China. The map shows Yantai and Weifang cities where mosquito samples were collected in 2018. The map was created using ArcGIS and does not involve any copyright issues. https://www.tianditu.gov.cn/.

A total of 3608 mosquitoes, comprising 5 species, were collected from 6 trapping locations in Shandong Province, China (Figure 1). Of these, 1674 were *Culex tritaeniorhynchus*, 514 were *Cx quinquefasciatus*, 195 were *Anopheles sinensis*, 1062 were *Aedes albopictus*, and 163 were *Armigeres subalbatus* (Table 1). *Culex tritaeniorhynchus* was the dominant species in the mosquito population collected in this study, followed by *Aedes albopictus*. From the collected mosquitoes, 372 male and 3236 female mosquitoes were identified. Male mosquitoes were discarded, and only female mosquitoes were retained, as they are the primary vectors of arboviruses due to their blood-feeding behaviour required for egg development [26]. Furthermore, these mosquitoes were categorized into 58 pools based on collection date, species, and location.

**Table 1.** Number of female mosquitoes collected by species and sampling site in Shandong Province, China, 2018.

Sampling sites	Mosquito species					Total
	*Culex* *tritaeniorhynchus*	*Aedes* *albopictus*	*Armigeres subalbatus*	*Cx quinquefasciatus*	*Anopheles sinensis*	
Weifang-Dazigou	0	414	0	0	0	414
Weifang-Fuyanshan	0	132	0	52	0	184
Weifang-Yujiashanqian	0	85	0	0	0	85
Weifang-Shantangcun	98	213	0	0	0	311
Yantai-chengguo	1352	0	0	247	101	1700
Yantai-wenchang	122	0	103	0	56	281
Yantai-yunfengshan	43	218	0	0	0	261
Total	1615	1062	103	299	157	3236

### Detection of WZSLV4 RNA in mosquitoes

qRT-PCR results indicated that WZSLV4 RNA was detected in 18 mosquito pools. The detection rate of WZSLV4 was 31.03% (the virus was detected in 18 of 58 mosquito pools). Among these positive pools, 11 pools were from *Culex tritaeniorhynchus* and seven pools were from *Aedes albopictus*, which were collected from four out of six trapping locations. The infection rate of WZSLV4 in *Culex tritaeniorhynchus* was 18.97% (11/58) and that of *Aedes albopictus* was 12.07% (7/58). No statistical difference was observed between the positive detection rates of the two types of mosquitoes (Table 2).

**Table 2.** Detection of WZSLV4 in mosquito pools by qRT-PCR, 2018.

Species	No. of mosquitoes	No. of pools (55–60 mosquitoes/pool)	No. Positive pools	Pooled infection rate (%)	*P*
*Culex tritaeniorhynchus*	1615	28	11	18.97	> 0.05
*Aedes albopictus*	1062	19	7	12.07	
*Armigeres subalbatus*	103	2	0	0	
*Cx quinquefasciatus*	299	6	0	0	
*Anopheles sinensis*	157	3	0	0	
Total	3236	58	18	31.03	

The last column is the *p* value for the comparison between *Culex tritaeniorhynchus* and *Aedes albopictus*.

### Isolation and growth properties of WZSLV4

The WZSLV4 18-LZ-22 strain was isolated from one pool of mosquitoes (C. tritaeniorhynchus). WZSLV4 RNA was detected in the supernatants of infected C6/36 cells, and the viral load began to increase on day 2, eventually reaching a plateau on day 12. No CPE was observed throughout the entire process of virus replication, even when virus replication reached the platform stage.

**Figure 2.**
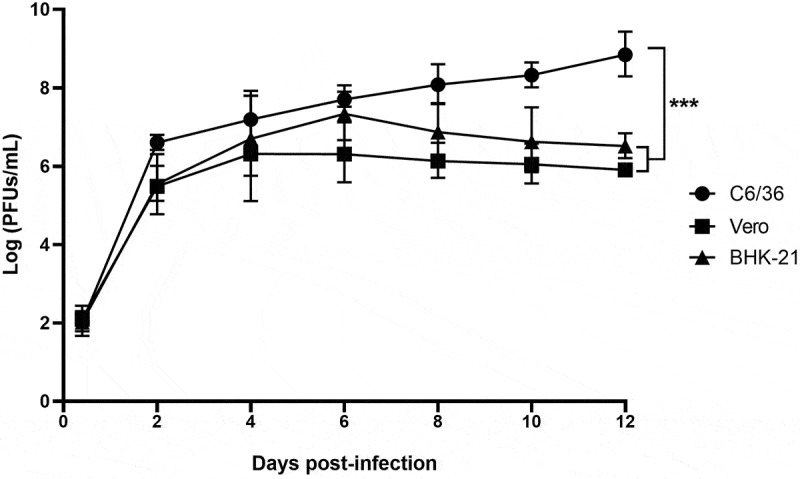
Growth kinetics of WZSLV4 strain 18-LZ-22 in different cell lines over 12 days post-infection. Viral RNA copies were quantified by qRT-PCR. Diamonds represent C6/36 cells, squares represent Vero cells, and triangles represent BHK-21 cells. Error bars represent standard deviation from triplicate experiments.

WZSLV4 was inoculated on Vero and BHK-21 cell lines to compare the growth characteristics of this virus in different cell lines. The results revealed that the virus copy numbers in the supernatants of infected Vero and BHK-21 cells increased slightly within 7 days, and no CPE was observed in either cell type. In contrast, the virus replicated more efficiently in C6/36 cells than in BHK-21 and Vero cells (Figure 2), the detailed logarithmic values of virus titres (PFUs/mL) at each time point are presented in Table 3. There was no difference in viral load between Vero and BHK cells, but there was a significant difference in viral load growth in C6/36 cells compared with the above two cells.

**Table 3.** Virus titre dynamics in C6/36, Vero, and BHK-21 cell lines at different post-inoculation times.

Time Post-Inoculation (Days)	Log C6/36-Virus Titre(PFUs/mL)		Log Vero-Virus Titre(PFUs/mL)		Log BHK-21Virus Titre(PFUs/mL)	
6 h	2.03	2.06	1.98	2.01	1.96	1.98
2	6.59	6.62	5.55	5.43	5.58	5.51
4	7.14	7.25	6.42	6.21	6.62	6.78
6	7.72	7.69	6.25	6.37	7.28	7.39
8	8.04	8.17	6.17	6.1	6.93	6.82
10	8.30	8.35	6.09	6.01	6.69	6.56
12	8.89	8.80	5.91	5.9	6.54	6.49

### Morphological features of virus particles

**Figure 3.**
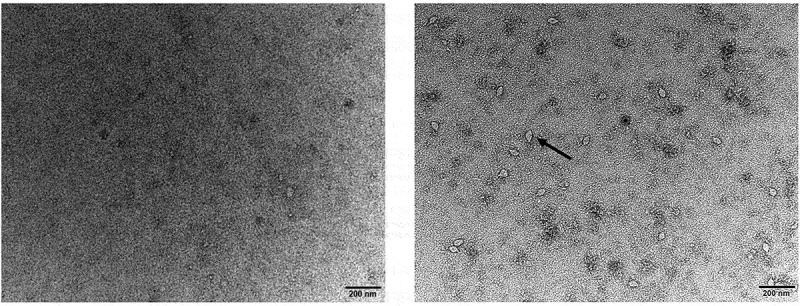
Electron microscopy images of WZSLV4 particles. Left panel shows negative control (uninfected C6/36 cells). Right panel shows WZSLV4 strain 18-LZ-22 particles (black arrows) with characteristic spindle shape, approximately 60 nm in length and 35 nm in width. Scale bar = 200 nm.

Viral particles without envelopes were observed in the supernatants of the infected C6/36 cells under an electron microscope. The particles were approximately 60 nm in length and 35 nm in width, and spindle-shaped structures were observed. These virions were evenly distributed in the field of vision (Figure 3) and were not detected in the control samples prepared from C6/36 cells.

### Complete viral genome organization

**Figure 4.**
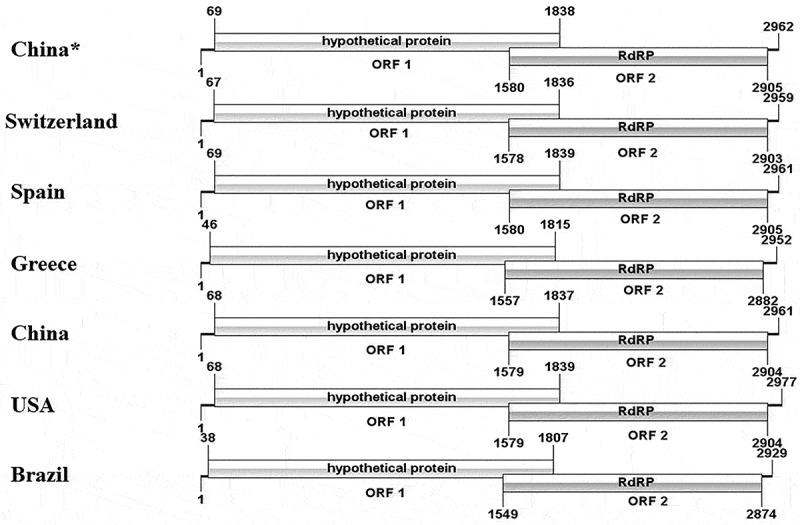
Genome organization and conserved domains of WZSLV4. Comparative analysis shows ORF1 (hypothetical protein) and ORF2 (RNA-dependent RNA polymerase) positions across strains from different countries. Asterisks indicate sequences from this study.

WZSLV4 was 2962 bp in length, with a G+C content of 45.07% (Figure 4). The genome contained two open reading frames (ORFs). ORF 1, which encodes the hypothetical protein, was located from nucleotides 69 to 1838. ORF 2 is an overlapping ORF encoded for the RNA-dependent RNA polymerase (RdRp), consisting of 441 amino acids. Further prediction of the sequences revealed that Peptidase S39 in ORF 1 had 377nt and RdRp in ORF 2 was 758nt. Peptidase S39 and RdRp were conserved domains for WZSLV4, even among Solemoviridae viruses. Moreover, three types of motifs were identified in ORF 2, providing elements for WZSLV4 replication and transcription. Compared with the whole-genome position of the ORF of WZSLV4 in other countries, the positions of the two ORFs in Greece and Brazil exhibited an alteration characterized by deletion that resulted in a shift of the reading frame. Our results showed that little difference existed among the motifs in the conserved regions of WZSLV4 nonstructural proteins from different countries, and the sequences of important protein-binding sites were conserved with almost no change. Additionally, NTP-binding sites and nucleic acid-binding sites were predicted in ORF 2.

### Phylogenetic analysis

**Figure 5.**
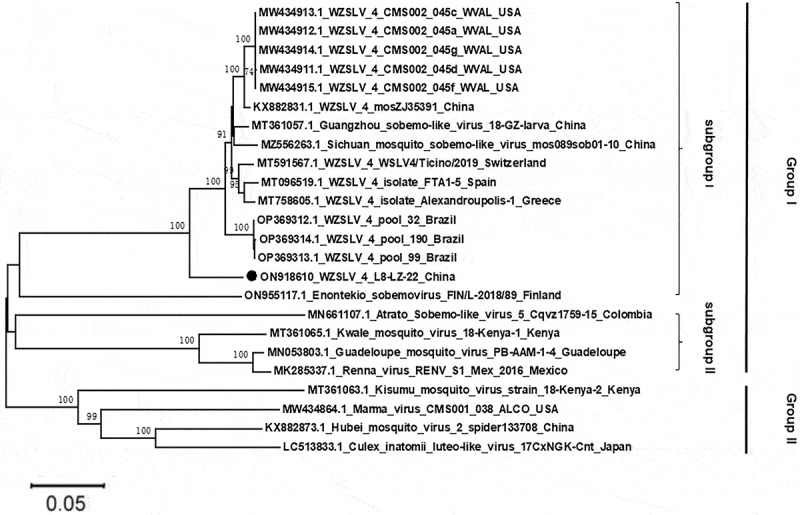
Phylogenetic analysis of complete WZSLV4 genome sequences. Neighbour-joining tree constructed with 1,000 bootstrap replicates. WZSLV4 strain 18-LZ-22 from this study is marked with a black circle. Bootstrap values > 70% are shown. GenBank accession numbers are provided for reference sequences.

Compared with the reported WZSLV4 genome information around the world, the WZSLV4 sequences obtained in this study were demonstrated to be similar to those found in Switzerland, Spain, Greece, China, the USA, and Brazil, with nucleotide identities of 92.57, 92.54, 92.55, 92.18, 92.07, and 91.03%, respectively. According to the results of evolutionary analysis, all WZSLV4 strains were divided into two groups (Figure 5). Group I primarily consisted of all WZSLV4 from different countries and the viruses related to WZSLV4, while group II comprised Kisumu mosquito virus from Kenya, Marma virus from the USA, Hubei mosquito virus 2 from China, and Culex inatomii luteo-like virus from Japan. In this study, within group I WZSLV4, the strains were identified as follows: two from China, five from the USA, one from Spain, one from Greece, one from Brazil, and one from Switzerland, which clustered together and formed subgroup I, along with Guangzhou sobemo-like virus, Sichuan mosquito sobemo-like virus, and Enontekio sobemovirus. The Atrato Sobemo-like virus, Kwale mosquito virus, Guadeloupe mosquito virus and Renna virus constituted subgroup II.

**Figure 6.**
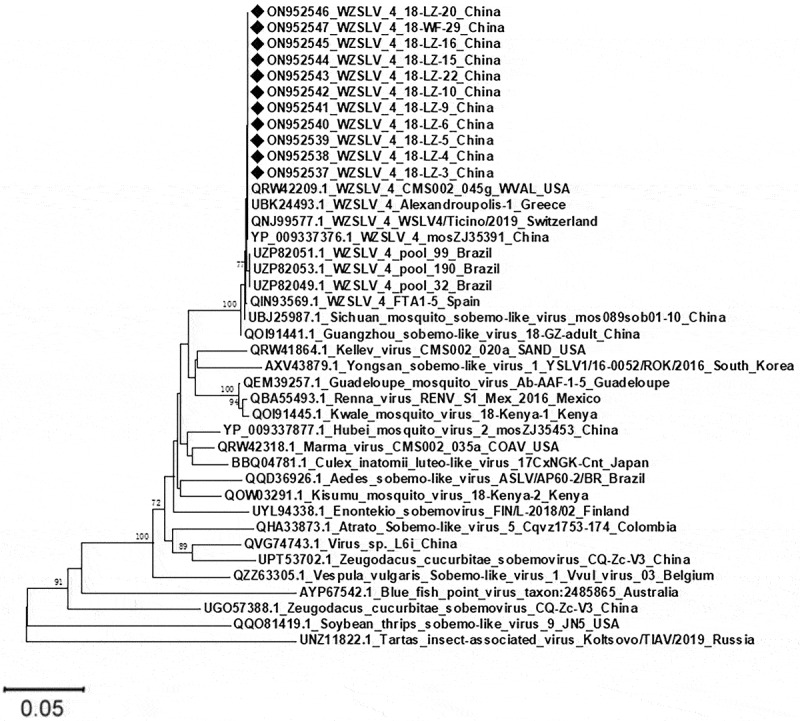
Phylogenetic analysis of partial WZSLV4 sequences (271 amino acids) from this study and reference strains. Neighbour-joining tree with bootstrap values > 70% shown. Black diamonds indicate sequences obtained in this study, demonstrating high similarity to global WZSLV4 strains.

In this study, the partial segment encoding hypothetical protein 2 of WZSLV4 was successfully amplified and sequenced from 11 out of 18 qRT-PCR-positive pools. Pairwise distance analysis indicated that these 11 sequences shared a nucleotide identity of 97.5–100.0%, suggesting that these sequences were closely related. A phylogenetic tree was constructed using an N-J approach based on 271aa sequences acquired in this study, along with WZSLV4-related sequences obtained from GenBank (Figure 6). Our results showed that All sequences obtained in our study clustered with WZSLV4 isolated from other countries. Furthermore, WZSLV4 was closely related to the Guangzhou sobemo-like virus and the Sichuan mosquito sobemo-like virus. However, WZSLV4 had rather distant phylogenetic relationships with the Tartas insect-associated virus and soybean thrips sobemo-like virus.

## Discussion

With the advancement of deep-sequencing technologies, an increasing number of novel disease-associated arboviruses have been discovered and genetically characterized [27]. WZSLV4 is a newly discovered non-segmented mosquito-borne RNA virus that shares a high identity with the Solemoviridae virus. The ongoing discovery of WZSLV4 in different mosquito species across diverse geographic regions has provided a theoretical basis for understanding its genomic structure and organization of WZSLV4. The host mosquito species and natural habitat of the WZSLV4 strain isolated in this study were similar to those of WZSLV4 strains from other countries. However, the genome sequence of the strain used in this study was slightly different from that of the known WZSLV4 strain. Compared with reported WZSLV4 genome sequences worldwide, the WZSLV4 sequence obtained in this study showed high nucleotide identities with strains from Switzerland (92.57%), Spain (92.54%), Greece (92.55%), China (92.18%), USA (92.07%), and Brazil (91.03%) [17–22]. These facts imply that the evolution and adaptation of WZSLV4 do not depend on its hosts and habitats.

Previous research has shown that *Aedes albopictus* is the object of WZSLV4 and was identified as the host [18–20]. However, our study showed that WZSLV4 could also be isolated from *Culex tritaeniorhynchus*. Therefore, it can be inferred that WZSLV4 exists in a variety of mosquitoes. WZSLV4 was first isolated in Shandong Province, demonstrating that WZSLV4 May be widely distributed in northeastern China and has formed a stable ecological cycle in the local area. Compared with the Tembusu Virus found in Shandong (64.7%) [28], the positive rate of WZSLV4 in mosquitoes was much lower (31.03%) in this study. This may be due to differences in sample sizes and sampling methods or the low carrying rate of WZSLV4 in mosquitoes in these local areas.

In the present study, the virus was isolated from *Culex tritaeniorhynchus* by culturing the C6/36 cells. The results showed that the cells were susceptible to the virus; in other words, it could promote the virus to complete its life cycle. Compared with C6/36 cells, Vero’s speed of reaching the peak of viral load in BHK-21 and Vero cells is slow. This shows that the virus can infect mammalian cells. A similar phenomenon has been previously observed for the Bustos virus discovered in mosquitoes, which does not significantly replicate in BHK-21 cells but in C6/36 cells [29]. In other words, WZSLV4 exhibited distinct replication profiles in different cells. Consequently, the infectious threat of the virus to humans requires related experimental proof. At present, it is not clear why WZSLV4 can proliferate slowly in mammalian cells without causing CPE. Further studies are needed to assess the biological and genetic determinants of the host range.

Similar to other non-enveloped viruses, the protein coat of the virus is not wrapped in a lipid membrane. Non-enveloped viruses have traditionally been thought to be released lytically as a result of cell death. However, recent evidence indicates that non-enveloped RNA and DNA viruses can egress without cell lysis. The manner in which the virus exits cells depends on the virus itself and the type of infected cell. In the absence of cell death, non-enveloped enteric RNA viruses can also exit the extracellular space through the membranous enclosure of progeny virus and non-lytic release via vesicles [30]. In this study, C6/36 cells were still not broken up after 20 days, leading to speculation that WZSLV4 was released in a quasi-enveloped form, similar to the hepatitis A virus (HAV) [31]. For the host and vector, this release method may reduce damage as much as possible.

The complete WZSLV4 genome from the direct NGS of qRT-positive mosquito pools was sequenced and analysed. The structures of the two ORFs are similar to those reported in previous studies. RdRp was involved not only in the genome replication process of an RNA from an RNA template but also in the encoding of some of the important proteins for the proper functioning and survival of viruses [32]. It was predicted in ORF 2 and considered to be a conserved sequence domain in WZSLV4. This demonstrated that RdRp in ORF 2 is a potent anti-WZSLV4 target. This discovery provided theoretical evidence for the in-depth study and exploration of WZSLV4.

Phylogenetic analysis demonstrated that the strain of WZSLV4, named 18-LZ-22, obtained in this study. An analysis of the branch length of the evolutionary tree indicates that our virus is most closely related to Sichuan mosquito sobemo-like virus (MZ556263.1) and Guangzhou sobemo-like virus (MT361057.1). Meanwhile, all strains of WZSLV4 were clustered together. This indicates that these strains possess high conservatism during species evolution under similar environmental conditions, or that they share a common virus ancestor. However, the nucleotide sequences of 18-LZ-22 had a remote relationship with those of the USA, possibly due to the evolutionary consequences of different WZSLV4 lineages or a wide range of mutations in the genome. Moreover, the 11 partial amino acid sequences of ORF 2 amplified in this study showed a high degree of similarity to all known WZSLV4 sequences. The principal reason for this may be that the ORF2 sequence of WZSLV4 was very conservative or that our amplified sequence was too short.

Thus far, the precise recombination mechanism and detailed evolutionary characteristics of the WZSLV4 genome have not been elucidated. As most arbovirus infections present with either no symptoms or only mild symptoms, these manifestations may also be overlooked or misdiagnosed, potentially leading to serious consequences. It is essential to conduct an artificial infection study on these mosquitoes to clarify whether they are vectors of diseases and assess their public health significance in WZSLV4 transmission.

This study has several limitations. First, the sampling scope was restricted to only two cities, Yantai and Weifang in Shandong Province. This limited geographical representation may not comprehensively reflect the distribution characteristics of WZSLV4 over a broader area, potentially missing out on diverse ecological niches where the virus could be present. Second, the mechanisms of viral replication in different cell types and host interactions remain uncharacterized. While WZSLV4 was shown to replicate in mammalian cells, key factors driving infection (e.g. receptor binding, immune evasion) and natural transmission routes (vertical/horizontal) require further investigation. Third, the functional studies of viral proteins in this research are currently based solely on bioinformatics predictions. There is a lack of experimental verification, such as enzyme activity assays and protein – protein interaction validations. Without these experimental data, it is challenging to deeply clarify the pathogenic mechanisms of the virus, as we are unable to precisely define the roles of these proteins in crucial processes like host cell invasion, viral genome replication, and virus particle assembly.

In conclusion, this study represents the first isolation of WZSLV4 from mosquitoes, and our findings showed that the virus obtained in our study had a high degree of identity to other strains from foreign countries at both the nucleotide and deduced amino acid levels. Moreover, WZSLV4 was detected in different species of mosquitoes in the study areas and could replicate in a variety of cells. Our results indicated that WZSLV4 has various host spectra and may pose a threat to animals and humans in this area.

## Data Availability

All nucleotide sequences were deposited in GenBank under the accession numbers ON918610, ON952537-ON952547. (https://www.ncbi.nlm.nih.gov/) The data that support the findings of this study are openly available in figshare at http://doi.org/10.6084/m9.figshare.26264543, http://doi.org/10.6084/m9.figshare.26264549, http://doi.org/10.6084/m9.figshare.26264588. The authors confirm that the data supporting the findings of this study are available within the article and its supplementary materials.

## Highlights

- WZSLV4 was firstly isolated from Shandong Province of China.
- One complete genome and 11 partial segments of WZSLV4 were sequenced and analysed.
- WZSLV4 was relatively conservative in evolution with two open reading frames (ORFs).

## References

[1] Xia H, Wang Y, Atoni E, et al. Mosquito-associated viruses in China. Virol Sin. 2018;33(1):5–11. doi: 10.1007/s12250-018-0002-9 29532388 PMC5866263

[2] Amaku M, Burattini MN, Coutinho FA, et al. Maximum equilibrium prevalence of mosquito-borne microparasite infections in humans. Comput Math Methods Med. 2013;2013:659038. doi: 10.1155/2013/659038 24454539 PMC3884616

[3] Kasman K, Ishak H, Alam G, et al. Resistance status of Aedes mosquitoes as dengue vectors and the potential of plant larvicides from Indonesia for biological control: a narrative review. Narra J. 2025;5(1):e1819. doi: 10.52225/narra.v5i1.1819 40352191 PMC12059870

[4] Stoler J, Al Dashti R, Anto F, et al. Deconstructing “malaria”: West Africa as the next front for dengue fever surveillance and control. Acta Trop. 2014;134:58–65. doi: 10.1016/j.actatropica.2014.02.017 24613157

[5] Kindhauser MK, Allen T, Frank V, et al. Zika: the origin and spread of a mosquito-borne virus. Bull World Health Organ. 2016;94(9):675–86c. doi: 10.2471/blt.16.171082 27708473 PMC5034643

[6] Rothan HA, Bidokhti MRM, Byrareddy SN. Current concerns and perspectives on Zika virus co-infection with arboviruses and HIV. Journal Of Autoimmunity. 2018;89:11–20. doi: 10.1016/j.jaut.2018.01.002 29352633 PMC5902419

[7] Bolling BG, Weaver SC, Tesh RB, et al. Insect-specific virus discovery: significance for the arbovirus community. Viruses. 2015;7(9):4911–4928. doi: 10.3390/v7092851 26378568 PMC4584295

[8] Fang Y, Li XS, Zhang W, et al. Molecular epidemiology of mosquito-borne viruses at the China-Myanmar border: discovery of a potential epidemic focus of Japanese encephalitis. Infect Dis Poverty. 2021;10(1):57. doi: 10.1186/s40249-021-00838-z 33902684 PMC8073957

[9] Uno N, Ross TM. Dengue virus and the host innate immune response. Emerging Microbes Infections. 2018;7(1):167. doi: 10.1038/s41426-018-0168-0 30301880 PMC6177401

[10] Silva LA, Dermody TS. Chikungunya virus: epidemiology, replication, disease mechanisms, and prospective intervention strategies. Journal Of Clinical Investigation. 2017;127(3):737–749. doi: 10.1172/jci84417 28248203 PMC5330729

[11] Waggoner JJ, Rojas A, Pinsky BA, et al. Yellow fever virus: diagnostics for a persistent arboviral threat. J Clin Microbiol. 2018;56(10). doi: 10.1128/JCM.00827-18 PMC6156298 30021822

[12] Musso D, Gubler DJ. Zika Virus. Clin Microbiol Rev. 2016;29(3):487–524. doi: 10.1128/cmr.00072-15 27029595 PMC4861986

[13] Fan N, Sun DW, Cheng R, et al. Isolation and identification of arbovirus in Hainan province, 2017–2018. Zhonghua Liu Xing Bing Xue Za Zhi. 2020;41(2):236–243. doi: 10.3760/cma.j.issn.0254-6450.2020.02.018 32164136

[14] Coffey LL, Forrester N, Tsetsarkin K, et al. Factors shaping the adaptive landscape for arboviruses: implications for the emergence of disease. Future Microbiol. 2013;8(2):155–176. doi: 10.2217/fmb.12.139 23374123 PMC3621119

[15] Yuan L, Huang XY, Liu ZY, et al. A single mutation in the prM protein of Zika virus contributes to fetal microcephaly. Science. 2017;358(6365):933–936. doi: 10.1126/science.aam7120 28971967

[16] Shi M, Lin XD, Tian JH, et al. Redefining the invertebrate RNA virosphere. Nature. 2016;540(7634):539–543. doi: 10.1038/nature20167 27880757

[17] Birnberg L, Temmam S, Aranda C, et al. Viromics on honey-baited FTA cards as a new tool for the detection of circulating viruses in mosquitoes. Viruses. 2020;12(3):274. doi: 10.3390/v12030274 32121402 PMC7150749

[18] Batson J, Dudas G, Haas-Stapleton E, et al. Single mosquito metatranscriptomics identifies vectors, emerging pathogens and reservoirs in one assay. Elife. 2021;10. doi: 10.7554/eLife.68353 PMC8110308 33904402

[19] Dovrolis N, Kassela K, Konstantinidis K, et al. ZWA: viral genome assembly and characterization hindrances from virus-host chimeric reads; a refining approach. PLOS Comput Biol. 2021;17(8):e1009304. doi: 10.1371/journal.pcbi.1009304 34370725 PMC8376068

[20] Kubacki J, Flacio E, Qi W, et al. Viral metagenomic analysis of Aedes albopictus mosquitos from southern Switzerland. Viruses. 2020;12(9):929. doi: 10.3390/v12090929 32846980 PMC7552062

[21] Andrade PS, Valença IN, Heinisch MRS, et al. First report of Wenzhou sobemo-like virus 4 in Aedes albopictus (Diptera: Culicidae) in Latin America. Viruses. 2022;14(11):2341. doi: 10.3390/v14112341 36366436 PMC9696862

[22] Xu Y, Xu J, Liu T, et al. Metagenomic analysis reveals the virome profiles of Aedes albopictus in Guangzhou, China. Front Cell Infect Microbiol. 2023;13:1133120. doi: 10.3389/fcimb.2023.1133120 37333852 PMC10272843

[23] Gangopadhayya A, Lole K, Ghuge O, et al. Metagenomic analysis of viromes of Aedes mosquitoes across India. Viruses. 2024;16(1):109. doi: 10.3390/v16010109 38257809 PMC10818685

[24] Syahidah D, Elliman J, Constantinoiu C, et al. Mosquito cells (C6/36) fail to support the complete replication of Penaeus merguiensis hepandensovirus. J Invertebr Pathol. 2017;145:31–38. doi: 10.1016/j.jip.2017.03.006 28315365

[25] Zhang Y, Li Z, Pang Z, et al. Identification of Jingmen tick virus (JMTV) in Amblyomma testudinarium from Fujian Province, southeastern China. Parasit Vectors. 2022;15(1):339. doi: 10.1186/s13071-022-05478-2 36167570 PMC9513871

[26] Alto BW, Juliano SA. Temperature effects on the dynamics of Aedes albopictus (Diptera: Culicidae) populations in the laboratory. J Med Entomol. 2001;38(4):548–556. doi: 10.1603/0022-2585-38.4.548 11476335 PMC2579928

[27] Roossinck MJ. Deep sequencing for discovery and evolutionary analysis of plant viruses. Virus Res. 2017;239:82–86. doi: 10.1016/j.virusres.2016.11.019 27876625

[28] Tang Y, Diao Y, Chen H, et al. Isolation and genetic characterization of a tembusu virus strain isolated from mosquitoes in Shandong, China. Transbound Emerg Dis. 2015;62(2):209–216. doi: 10.1111/tbed.12111 23711093

[29] Fujita R, Kuwata R, Kobayashi D, et al. Bustos virus, a new member of the negevirus group isolated from a mansonia mosquito in the Philippines. Arch Virol. 2017;162(1):79–88. doi: 10.1007/s00705-016-3068-4 27671777

[30] Owusu IA, Quaye O, Passalacqua KD, et al. Egress of non-enveloped enteric RNA viruses. J Gen Virol. 2021;102(3). doi: 10.1099/jgv.0.001557 PMC8515858 33560198

[31] Wang M, Feng Z. Mechanisms of hepatocellular injury in hepatitis a. Viruses. 2021;13(5):861. doi: 10.3390/v13050861 34066709 PMC8151331

[32] Pathania S, Rawal RK, Singh PK. RdRp (RNA-dependent RNA polymerase): a key target providing anti-virals for the management of various viral diseases. J Mol Struct. 2022;1250:131756. doi: 10.1016/j.molstruc.2021.131756 34690363 PMC8520695

